# Analysis and study of the potential increase in energy output generated by prototype solar tracking, roof mounted solar panels

**DOI:** 10.12688/f1000research.27641.1

**Published:** 2020-11-30

**Authors:** Jacek Harazin, Andrzej Wróbel

**Affiliations:** 1 Departament of Engineering Processes Automation and Integrated Manufacturing Systems, Politechnika Śląska, Gliwice, Śląskie, 44-100, Poland

**Keywords:** solar tracker, household solar energy, roof solar panels

## Abstract

Roof mounted solar panels come in form of fixed panels, unable to adjust to sun’s position during day and throughout the year. As an effect, the efficiency of such solution is usually dependent on the roof slope and position of the building in relation to sun’s day arc during seasons. These problems can be bypassed in free standing solar installations by equipping solar panels with solar tracker installations. Thanks to solar tracking, solar panels can be dynamically positioned perpendicular to the sun position and gather energy more efficiently throughout the day. This article presents a possibility of creating a roof mounted solar tracking panel to increase its efficiency. A prototype of solar tracking panel with two axes of movement was designed with an intention of an easy adaptation to being mounted on sloped surfaces of building roofs. A reference stationary panel was used to compare the efficiency of both solutions. A 5-day study was carried out to determine if the proposed solution could provide any benefits. Based on the study, the authors made an attempt to draw a conclusion whether the design could considerably increase the solar energy output to be worth the extra spending associated with solar tracker installation.

## Introduction

Renewable energy sources are increasingly perceived by the public as a beneficial alternative to fossil fuels in context of preservation of the environment by limiting the carbon emissions. Recent decades show a steady increase of public interest in percentage of renewable energy sources in the overall energy production ^1– 7^. More people are also choosing to supply their homes with renewable energy, mainly through the installation of household solar panel systems in the form of free-standing solar installations and roof mounted panels. Even the citizens of countries located in higher latitudes (such as Poland, England or Sweden, and other countries in Europe located on similar latitudes) with less annual access to solar energy due to weather and seasons have showed an increase of interest in household solar panel installations. The increase of awareness and willingness to focus on renewable energy sources can partly be contributed to numerous government programs that promote the transition with numerous discounts and advertise positive results of such transition. The EU is putting large emphasis on promoting renewable energy sources in recent years by dedicating numerous grants for co-financing own solar installations and introducing regulations that favor renewable energy. Thanks to the financial support it is possible to fund a household solar installation with relatively small financial outlay which attracts many household owners previously discounting such solution due to large entry costs. Another factor that contributes to this situation is the advancement in solar cell efficiency and energy storage technologies. This further decreases the payback time for investment costs which attracts more people.

Depending on the availability of open space, future solar installation owners decide either on purchasing big or small factor free-standing solar panel installations or roof-mounted solar panel installations. Apart from the space availability, authors in 5– 7 also mention financial factors such as estimated monthly and yearly household power consumption or costs related to solar panel installation and maintenance. Other factors are connected with country regulations regarding the use of renewable energy sources such as discounts and grants for installations, handling of the energy surplus (whether an owner is obligated to return any surplus of energy into the network and if it’s then available as a buffer or if there is any financial gain to be had). Another set of factors is related to weather conditions in a region where the new solar installation is to be mounted. The change in environment albedo and snow/rain fall are some of the more important factors as snow coverage during winter season can significantly alter albedo, resulting in an increase of solar panel energy output. On the other hand, snow coverage of the panels themselves or rainy weather can seriously hinder any power generation in contrast. One of the most important factors in case of stationary solar panels is the setting of optimal tilt angle for maximally efficient power production of solar panels throughout the year. The authors in 8, 9 determine the optimal tilt angle based on slightly different factor compositions. The authors in 8 prioritized maximization of the average solar irradiance throughout the taken period whereas the authors in 9 also taken maximum revenues in the area of solar panel into account in their algorithm. As a result, the optimal tilt angle can vary depending on the emphasis taken on the period when solar panels work (whole year, emphasis on the summer season or skipping of the winter season) and other economic factors that can be considered.

The advantage of free-standing solar installations over the roof mounted ones can become apparent at the point of determining the optimal tilt angles. Free-standing solar panels can be easily equipped with solar tracking devices, coupled with one or two motors that rotate the panel in one or two axes of movement. This method eliminates the need for determining a fixed tilt angle and allows for dynamic adjustment of solar panel tilt angle depending on the season and time of day. The difference in energy production efficiency between stationary mounted solar panels and solar panels equipped with solar tracking capabilities can vary from 10% to 60% depending on the tracking technology used and considered time of the day ^7, 10– 19^. In addition, countries that are located on higher geographic latitudes can get more benefit from tracker installations because of the higher volatility of sun position during different seasons. The downside, however, is the lower return in additional costs associated with solar tracker and motors required for the system. The cost efficiency of different solutions may vary greatly, depending on whether there are policies in effect, that allow for either storing excess energy in the communal grid or selling it back to the grid ^7^. If there are no such policies in effect, the use of two axis solar trackers may be the only option worth considering due to high amount of budget that has to be allocated for batteries that store the excess energy. If such policies exist, then solar trackers appear more cost effective, especially on areas with lower solar potential.

This project aims to create a prototype solution that would enable solar tracking on panels usually mounted on household roofs in urbanized areas. Such a solution could potentially increase the daily energy output of solar panels, giving better energy yields from a relatively small space of a household roof. Such a solution could also make roof solar installations more viable in areas of the globe, where daily sun coverage is mediocre or poor. This article is focused on the testing of the first few iterations of the prototype to verify the authors' prediction that such a project could bring benefit.

## Methods

### Object under examination

**Figure 1.**
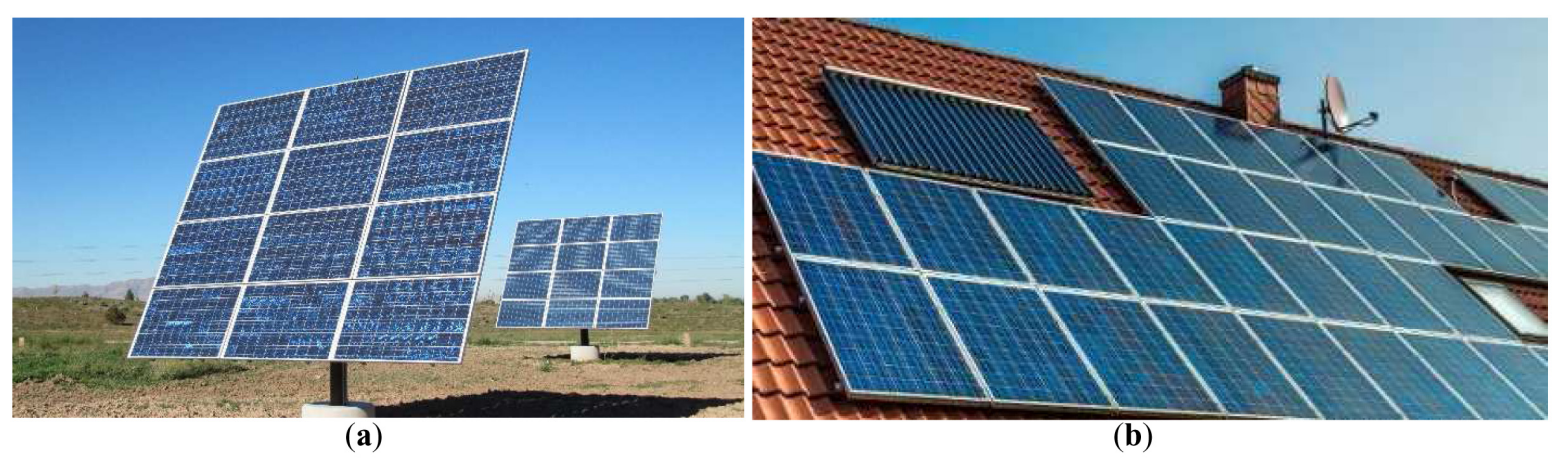
Examples of different types of solar installations. ( **a**) Free standing solar panel, equipped with a solar tracker and actuators, enabling the tracking of sun position throughout the days and seasons ^19^. ( **b**) Roof-mounted solar panel with a fixed mount and no ability to track the sun.

This article concentrates on exploring possibilities of combining the flexibility of free-standing solar panels, equipped with solar trackers and the relatively compact nature of roof mounted solar panels ( Figure 1). The idea is motivated by the search for a middle ground solution that would provide at least a portion of benefits carried from the use of solar tracking panels without the large space requirement. Building owners in heavily urbanized areas and household owners in tightly packed residential districts do not possess enough space to fit a cost-effective, ground-mounted solar installation, large enough to justify being fully equipped with solar trackers. The article has a preliminary character in terms of tackling many aspects of solar installations and will be mainly focusing on potential gains in power generation efficiency that come with solar tracking installations. The design of the test stand will be oriented towards the ability to be mounted on a sloped roof. A study will involve an analysis of the solar tracking panel efficiency versus a fixed solar panel in a configuration resembling a mounting on a sloped roof. The obtained results will serve in further research to determine if the potential gain is enough to justify expenses that relate to solar tracking installations.

### Measuring equipment and appearance of the laboratory stand

**Figure 2.**
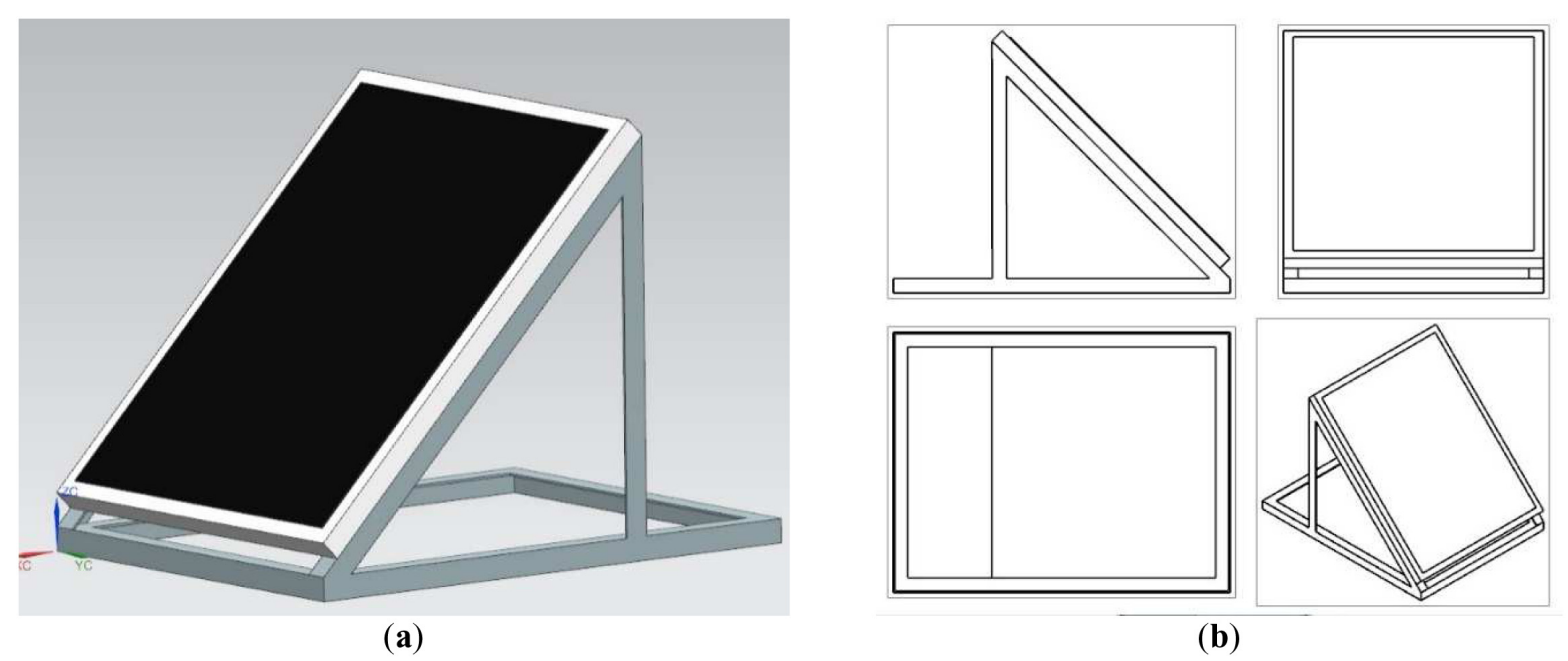
CAD model of a test bench for a reference solar panel mounting. ( **a**) Isometric view. ( **b**) Spatial projections.

The plan for the study included construction of two test benches after conceptual design phase in SIEMENS NX 12. The first test bench was meant for creating a reference data set for the standard mounted roof solar panel. It was designed with the slope angle of 45° and a set of adjustable feet to give it some room for adjustment of the slope on site. The model and its individual spatial projections were shown of Figure 2.

**Figure 3.**
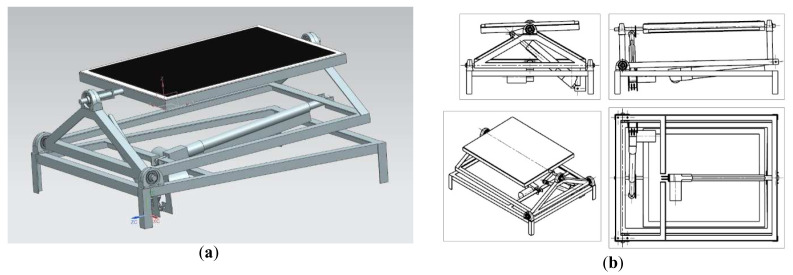
CAD model of a test bench for a prototype roof mounted solar panel equipped with a solar tracker. ( **a**) Isometric view. ( **b**) Spatial projections.

The second test bench was capable of rotating in two axes. In order to enable the following of the sun trajectory during the day, a GPS-based solar tracker was added. The assumption was to enable vertical tilt of around 90 degrees and horizontal tilt of roughly 45° in each direction. To realize the movement, bench was equipped with two self-locking linear actuators, with substantial force of 4000 N to provide control even in windy weather. In order to make the construction more compact and suited for roof applications, the decision was made to use the vertical axis as primary axis. The horizontal axis of movement was a secondary axis. This approach unfortunately restricted the freedom of movement, of the solar panel and complicated the solar tracking algorithm because of the necessity to convert spherical coordinate system to a cylindrical one, in case of the azimuth angle and panel horizontal tilt. The solar tracker was created using an Arduino Uno programmable circuit, coupled with a GPS module, with a solar tracking algorithm that uses GPS as input data. After a search for appropriate solar tracking algorithm in literature sources, a decision was made to use the derivative of a PSA (Plataforma Solar de Almeria) algorithm to calculate sun position out of the data provided by the GPS module ^10– 14, 17, 20– 23^. The model of a solar tracking panel and its individual spatial projections were shown on Figure 3.

**Figure 4.**
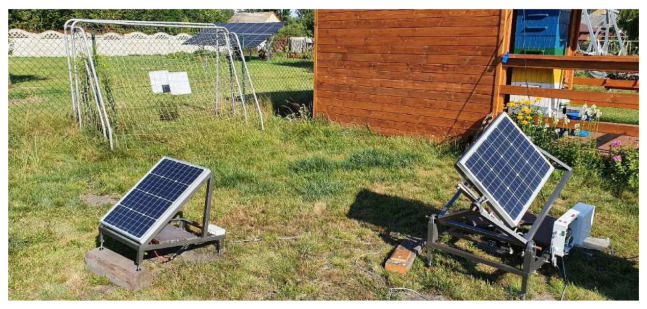
Completed setup placed at its final study destination. The stationary solar panel was raised to adjust its angle to around 30° which is an optimal angle for the latitude of Poland, Upper Silesia, based on the information provided by local solar panel mounting companies.

To accommodate the aforementioned system of axis movement with the applied algorithm, a series of angular conversions was prepared, that converted outputted azimuth and zenith angles into angles that were to be achieved by the stand, to properly track the sun position. Solar tracker was set to adjust panel position every 90 seconds. In terms of a proper comparison between results obtained from the reference test bench and the solar tracking test bench, both of them were equipped with identical 50 W monocrystalline solar panels, with a work surface of 540 × 670 mm, capable of outputting 2.78 A of current at 18 V. The same Arduino Uno controller used for the implementation of the solar tracker algorithm was also responsible for registering power outputs of both solar panels at 30 second intervals due to lack of a proper MPPT controller. The power readings were saved on the SD Card memory in form of files that contained consecutive power readings in beforementioned 30 second intervals recorded throughout the day. The outputted power was being drained by two 100 W power resistors, converting all the electrical energy into heat. Each panel had its own power registering loop, coupled with own power resistor. The circuitry involved in the study is completely separated from the measurement loop to avoid any interference from the actuators or the controller. To avoid overflow of memory on the Arduino Uno controller it was unfortunately decided to omit the measurement of power draw from the whole solar tracker circuit during its operation. It is a consideration to upgrade the measuring system in the future experiments, to also include this power draw in the equation. Although the panels are planned to be implemented as a new roof mounted solution, it was very hard to find building owners willing to spare their roof for testing purposes. To mitigate this obstacle, a decision was made to try and consider the possible range of movement, a panel could have, when mounted on a roof. The possible application of the current construction to a building roof would involve rotating the panel by 180° along its base and adjusting the calculation of zenith angle, to include roof inclination in the equation. For the purposes of this experiment, both panels were placed on the ground with adjustable feet as supports. The mechanical part of both test stands was completed around June. The electrical wiring and programming of the Arduino controller was completed in August, just before the tests began. A complete setup placed in its final study destination can be seen on Figure 4.

### Test procedure

The research was conducted right after the completion of test benches which took place in August 2020. It took a total of 5 days between 14.08.2020 and 18.08.2020 (including that day). The research was conducted in Poland, Upper Silesia, with geographic coordinates of 50°22' N and 19°15' E. The sunrise, at the time of the experiment, on average, took place at 5:33 CEST (3:33 GMT). The sunset, at the time of the experiment, on average, took place at 19:58 CEST (17:58 GMT). The average length of the day was around 14 hours and 35 minutes. The area at which the experiment was conducted, was a rectangular plot with width of around 15 meters (in W-E direction) and length of around 100 meters (in S-N direction). Test benches were placed on the furthest side towards the west of the plot and around the middle in the N-S direction. The positioning was dictated by the trees that were growing in the near vicinity of the plot, from the east side. Because of the aforementioned trees, the sun was accessible from around 7:30 CEST (5:30 GMT) in terms of mornings. The view was almost unobstructed from the west side, however, which allowed for tracking the sun almost up to the time of sunset. Both test stands were placed on adjustable feet to keep them in level. According to the information provided, the procedure of daily measurement was as follows:

• taking off the covers that secure the stands from the morning dew and rain
• activation of test stands around 7:00 CEST (5:00 GMT) and their calibration
• verification of correct solar panel alignment after the calibration and routine check of the state of hardware and software
• in case of any errors or damage spotted, necessary debugging or repairs
• continuation of the experiment throughout the day, up until around 20:30 CEST (18:30 GMT) when the sun is below the horizon already
• stopping the research aperture by cutting the circuitry from external power supply
• extraction of the gathered data by pulling the SD card with the data from the SD card module and copying the files to the laptop and USB stick for redundancy
• securing the stand with covers for the night

The raw data consisted of the date, at which each measurement series was done, time at which each sample was taken and power readings from the fixed, reference solar panel and the tracking panel. The data from each day was processed in Microsoft Excel. All measurements taken throughout the period of the study were placed on a uniform timescale (to achieve that, some samples had to be moved in time by 1–10 seconds). The timescale ranged from 7:00 CEST (5:00 GMT) to 20:00 CEST (18:00 GMT) reflecting the daily study schedule. The time interval between each sample taken was 30 seconds.

## Results

Table 1 and Table 2 show the aggregated data from gathered samples, showing the total energy gathered on each day by stationary and tracking solar panel. See *Underlying data* for the full combined and ordered data collected from each sample ^24^.

Table 3 shows a difference in energy gathered between tracking panel and a stationary panel, derived from the data. Table 4 shows the calculated efficiency of tracking solar panel in reference to stationary solar panel. The data were grouped by readings taken from each day of the study period. The day was also divided into three periods: morning (from 7:00 to 10:00), midday (from 10:00 to 15:00) and evening (from 15:00 to 20:00). Throughout the study period, the measurements were taken during different weather conditions. The weather was mostly sunny from 14th to 17th with occasional cloud patches covering the sky from few minutes to about an hour and a short rainfall occurring during the evening of 16th. There was a substantial cloud coverage throughout the evening of 17th.

**Table 1.** Energy collected by stationary solar panel.

Date	Energy collected, J			
	MORNING	MIDDAY	EVENING	TOTAL
14.08.2020	47491.50	559920.30	165526.80	772938.60
15.08.2020	66753.30	418826.40	141862.50	627442.20
16.08.2020	119195.40	772084.80	99255.90	990536.10
17.08.2020	134105.40	638697.60	50764.80	823567.80
18.08.2020	61661.10	3594.30	-----	65255.40
TOTAL	429206.70	2393123.40	457410.00	3279740.10
AVERAGE	85841.34	478624.68	91482.00	655948.02

**Table 2.** Energy collected by tracking solar panel.

Date	Energy collected, J			
	MORNING	MIDDAY	EVENING	TOTAL
14.08.2020	11335.20	564304.50	311209.80	886849.50
15.08.2020	54188.10	453057.00	404502.60	911747.70
16.08.2020	194316.60	802172.40	149549.10	1146038.10
17.08.2020	204126.00	662093.70	109598.70	975818.40
18.08.2020	106896.30	4080.90	-----	110977.20
TOTAL	570862.20	2485708.50	974860.20	4031430.90
AVERAGE	114172.44	497141.70	194972.04	806286.18

**Table 3.** Difference in energy collected between stationary and solar tracking panels.

Date	Difference in energy collected, J			
	MORNING	MIDDAY	EVENING	TOTAL
14.08.2020	-36156.30	4384.20	145683.00	113910.90
15.08.2020	-12565.20	34230.60	262640.10	284305.50
16.08.2020	75121.20	30087.60	50293.20	155502.00
17.08.2020	70020.60	23396.10	58833.90	152250.60
18.08.2020	45235.20	486.60	-----	45721.80
TOTAL	141655.50	92585.10	517450.20	751690.80
AVERAGE	28331.10	18517.02	103490.04	150338.16

**Table 4.** Efficiency gain of tracking panel in reference to stationary panel.

Date	MORNING		MIDDAY		EVENING		TOTAL	
	RMI	CIG	RMI	CIG	RMI	CIG	RMI	CIG
14.08.2020	-83.57%	-76.13%	0.82%	0.78%	87.77%	88.01%	12.64%	14.74%
15.08.2020	-16.27%	-18.82%	8.2%	8.17%	184.77%	185.14%	44.62%	45.31%
16.08.2020	62.74%	63.02%	3.91%	3.9%	50.45%	50.67%	15.7%	15.7%
17.08.2020	52.1%	52.21%	3.68%	3.66%	115.78%	115.9%	18.49%	18.49%
18.08.2020	73.19%	73.36%	13.94%	13.54%	-----	-----	69.89%	70.07%
TOTAL	28.68%	33%	3.87%	3.87%	112.8%	113.13%	22.26%	22.92%
AVERAGE	31.57%	33%	4.01%	3.87%	113.17%	113.13%	21.94%	22.92%

RMI, raw measurement inputs; CIG, calculated interval gains.

Due to a storm occurring after the morning of 18th the test, that day, had to be stopped. Additionally, in the morning of 14th and 15th there was a necessity to do some maintenance of the solar tracker which involved debugging the software and resulted in a loss of potential power gains during this time period. It can be seen in the negative values, presented on Table 3 and Table 4. This big difference was caused by the tracking panel being greatly misaligned to the position of the sun. Also, in the Table 4, two separate percentages were listed and called raw measurement inputs (RMI) and calculated interval gains (CIG). Because of the way, the data was being gathered (30 second intervals), to calculate the energy produced between each measurement, a mean value between 2 subsequent measurements was taken and multiplied by the time between each measurement. This method is unfortunately prone to rounding errors and that is why the calculation of efficiency was done through the comparison of both RMI and CIG. Deviations between those two percentages are especially apparent in mornings of 14 ^th^ and 15 ^th^ when there were troubles with the operation of solar tracker.

**Figure 5.**
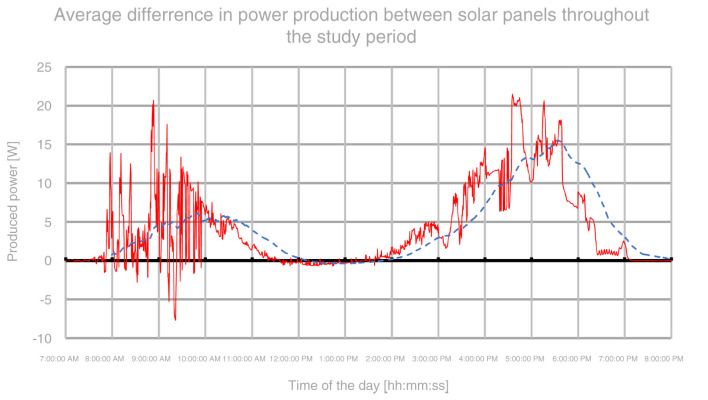
Graph representing the daily difference in power gains between panels during the study.

The aggregated data showed a rough increase in tracking panel power generation of about 22–23% in relation to the stationary panel throughout the study period. In terms of mornings, the data was heavily disturbed by the problems that occurred during the test. Nonetheless, the data shows an increase in power income by 29–33% during morning periods throughout the entire study. Evening periods showed over a double amount of energy produced whereas middays showed about 4% increase due to both panels working at full capacity. A graph, presenting an average difference in power gains between stationary and tracking solar panel, throughout the day, was shown on Figure 5.

Figure 5 represents an average daily difference in consecutive power output readings between stationary and solar tracking panels based directly on gathered samples from both solar panels throughout the study period. The red line represents an average of measurements taken from the same time of day throughout the study period. Again, due to problems in the first two days of the study, the graph is heavily disturbed in the morning period. The blue dashed line represents a moving average with a window of 120 samples (equal to one hour). Judging by the moving average, it can be noticed that the peak gain of around 5 W/s was achieved between 9 and 10:30. The efficiency gains are diminishing past 11 and there are no visible gains between 12 and 14. Again, past 14, the solar tracking panel starts to gather more energy with a peak gain of around 15 W/s around 17:30. After that, the gains start to quickly diminish again to 0 towards the evening hours.

## Discussion

Results of the study have shown an average daily power output of a stationary solar panel at a level of 0.182 kW-h per day and an average power output of solar tracking panel at a level of 0.224 kW-h per day. The difference in outputted power between stationary and solar tracking panel was 0.042 kW-h which amounts to a 22–23% gain in panel efficiency due to solar tracking ability. The overall mediocre performance of both solar panels over the entire span of the study can be attributed partly to some cloud coverage in the 3 ^rd^ and 4 ^th^ day of the study, and a thunderstorm in the 5 ^th^ day, which forced the authors to terminate the study as early as 10 am. Gains in tracking solar panel performance of this design are lower than those presented in 7, 12 and comparable to results presented in 16. The short study period of just 5 days in this setting is highly prone to statistical errors due to the small amount of data collected. Technical difficulties with solar panels in the beginning of the study and bad weather towards last days could have also contributed to this poor performance result over other studies.

There is a noticeable gain in power output efficiency of solar tracking panel during the morning (29–33%) and especially evening hours (113%). It is most probably due to stationary solar panels positioning and tilt being optimized for maximum efficiency during hours with best solar irradiance. Because of that, there are almost no gains of power over stationary solar panel during midday hours. The daily average collective gain in power from morning and evening hours with a solar tracking panel amounted to around 0.037 kW-h, which contributes around 88.1% of total power gains of solar tracking panel over the stationary solar panel. The results could’ve probably been even higher in favor of mornings and evenings if the test period took longer. This means that tracking solar panels take most of their benefit from solar energy collection during mornings and evenings. Given that the sun elevation decreases significantly towards the winter season, it is possible that more gains are to be had.

It is very hard to determine the economic cost efficiency based on the information gathered so far. Solar panels bought for the study cost around 393 PLN (101.91 USD) each and the spending associated with construction of both stands reached around 1700 PLN (440.73 USD), with about 2/3 of this cost being spent on the tracking solar panel because of its increased complexity. Another 1030 PLN (267.02 USD) was associated directly with solar tracking panel and that was a spending on actuators. Around 3250 PLN (842.53 USD) was spent in total on the construction of both test stands from which around 2556 PLN (662.61 USD) was associated with the construction of solar tracking panel. The solar tracking panel took around 78% of the budget and 22% were associated with the stationary panel. This means that solar tracking panel project cost around 3.7 times more than the stationary solar panel which indicates a fairly high cost compared to gains in efficiency. However, these costs are uncertain due to few factors. It has to be noted that this project was done by the researchers themselves omitting any costs associated with professional assembly and installation which influence the cost of solar panel installation significantly as pointed out in 5. Also, due to small factor of the study, there were no batteries or inverters involved which also contribute greatly to the costs of overall installation ^5, 7^. Broader research should be conducted to determine the effects of abovementioned factors on the overall economical profitability of such installation, including any grants and discounts or national policies regarding renewable energy.

## Conclusions

Despite the big number of obstacles and troubles during the study period, the obtained results show some correlation with results presented in other papers. This signifies that such adaptation of roof mounted solar panels may become a valid solution. The obvious conclusions from this study involve the need for longer testing periods to increase the data pool and reduce the effect of errors and anomalies on the aggregated data. The 50 W power generation limit of used solar panels should also be increased in the future by upscaling the test stands, to investigate power increase with more photovoltaic surface area and decrease the margin of error that is contributed to small scale experiments. Future studies should also involve studying periods in other seasons, to investigate potential power gains that come with different sun elevations during different seasons. The tracking algorithm used for this study was very basic and lacked many utility functions that a fully-fledged solar tracker has. This is an issue that should also be addressed before future studies. For longer study periods, the tracker must be upgraded with a wind measuring probe that is able to determine bad conditions for solar panel work and be able to fold the panel, to prevent any damage. Also, a data registering system must be upgraded with additional memory banks, to be able to collect more data during long periods and provide the data on the power draw, generated by the circuitry and the actuators. Alternatively, a proper MPPT power draw registering system must be added to the testing stand to properly measure the power draw of all elements. It is also very important to investigate the economical profitability of the proposed solution by investigating economical landscape in search of additional costs and benefits associated with solar panel installation.

Given the infancy of this project, this study was able to provide the data needed for its continuation. In further studies, it is imperative to thoroughly analyze all the potential scenarios that this system would be able to work with, such as different weather conditions and different seasons. Furthermore, a problem of power draw, by the included circuitry, and its effect on the resulting efficiency should be investigated. Lastly, it is also important to conduct a study referring to the advantages of using solar trackers on roof mounted solar panels versus the additional costs that they can generate and potential decrease of roof space that is connected with the problem of panels covering each other.

## Data availability

Mendeley Data: Research data on prototype solar panel power output. http://doi.org/10.17632/2bgvhmtrpx.1 ^24^.

This project contains the following underlying data:

• Samples. (A collection of data recordings in the .txt format from the days of the study.)
• Research_data.xslx. (Spreadsheet with combined and ordered data collected from each sample.)

Data are available under the terms of the Creative Commons Attribution 4.0 International license (CC-BY 4.0).
