# Supplementary material for: Analysis and study of the potential increase in energy output generated by prototype solar tracking, roof mounted solar panels
Source: F1000Res. 2022 Jul 18;9:1381. Originally published 2020 Nov 30. [Version 2] doi: 10.12688/f1000research.27641.2 (PMC9478504; doi:10.12688/f1000research.27641.2)
Supplement: Peer review history

# Peer review: Reviewer response for version 2

**DOI:** 10.5256/f1000research.136114.r144534

**Published:** 2022-09-15

**Authors:** Zeundjua Tjiparuro

**Affiliations:** 1 Department of Mechanical, Energy & Industrial Engineering, Botswana International University of Science and Technology, Palapye, Botswana

The second version of the article is accepted as presented and recommended for indexing.

Is the work clearly and accurately presented and does it cite the current literature?

Partly

Are all the source data underlying the results available to ensure full reproducibility?

Partly

Is the study design appropriate and is the work technically sound?

Partly

Are the conclusions drawn adequately supported by the results?

Partly

Are sufficient details of methods and analysis provided to allow replication by others?

Partly

Reviewer Expertise:

NA

I confirm that I have read this submission and believe that I have an appropriate level of expertise to confirm that it is of an acceptable scientific standard.

# Peer review: Reviewer response for version 1

**DOI:** 10.5256/f1000research.30552.r119191

**Published:** 2022-02-28

**Authors:** Wan Zuha Wan Hasan

**Affiliations:** 1 Department of Electrical and Electronic Engineering, Faculty of Engineering, Universiti Putra Malaysia, Selangor, Malaysia

The prototype solar tracking was well working based on the proposed solution. It was a very practical solution to provide a solar tracker in order to increase power energy from solar. The outputs have shown very significantly. Since the solar tracker is a quite well-known solution, the author should discuss more on installation and costing issues against the total collected energy for both ground and roof top installation.

Is the work clearly and accurately presented and does it cite the current literature?

Yes

If applicable, is the statistical analysis and its interpretation appropriate?

Yes

Are all the source data underlying the results available to ensure full reproducibility?

Yes

Is the study design appropriate and is the work technically sound?

Yes

Are the conclusions drawn adequately supported by the results?

Yes

Are sufficient details of methods and analysis provided to allow replication by others?

Yes

Reviewer Expertise:

Robotic and Automation, Solar Energy

I confirm that I have read this submission and believe that I have an appropriate level of expertise to confirm that it is of an acceptable scientific standard.

# Peer review: Reviewer response for version 1

**DOI:** 10.5256/f1000research.30552.r79908

**Published:** 2021-03-11

**Authors:** Zeundjua Tjiparuro

**Affiliations:** 1 Department of Mechanical, Energy & Industrial Engineering, Botswana International University of Science and Technology, Palapye, Botswana

1. In the abstract please edit the line: *This article presents a possibility of creating a roof mounted solar tracking panel to **increase its efficiency***. (Change bold words to…to increase irradiance efficiency).
2. In the Introduction please edit this line: *The increase of awareness and willingness to focus on renewable energy sources can partly be **contributed*** …(change bold word to: attributed).
3. Still in the Introduction, please edit this line: *Thanks to the financial support it is possible *(..punctuate with a coma after the word support).
4. In paragraph 2 of the Introduction in the sentence [ *big or small factor*…. (delete the word factor)].
5. Sentence 2, in paragraph 2, of the Introduction: is either misplaced, or incomplete and will need editing to make it fit where it has been placed. The main problem is that it combines several factors which are diametrically opposite. For instance, space availability is certainly a limitation, but then combining this with household power consumption, which is certainly a motivation for installing solar, makes the write up sound ungrounded. Then another deterrent follows in: costs of installation and maintenance. This how your write up looks starting with the sentence: *Apart from the space availability, authors in 5–7 also mention financial factors such as estimated monthly and yearly household power consumption or costs related to solar panel installation and maintenance*….[the question is, the factors are mentioned as what? Problems to the installation or factors influencing installation?] But the sentence that follows is one for motivating for installation, but the next two on albedo and snow are a mix of opposites again, leaving the reader confused as to whether the author is motivating or discouraging. In fact, most of the things that the author wants to write about here have been covered in the preceding paragraph, and he is better off deleting the whole of this paragraph.
6. The reader will be lost as to which of these installations are better in this sentence: [ *The difference in energy production efficiency between stationary mounted solar panels and solar panels equipped with solar tracking capabilities can vary from 10% to 60% depending on the tracking technology used and considered time of the day*].
7. This sentence is not fitting well where it has been placed [ *In addition, countries that are located on higher geographic latitudes can get more benefit from tracker installations because of the higher volatility of sun position during different seasons*] since the preceding sentence is on the advantage of free standing installations being amenable to fitting trackers, the above sentence comes in to disrupt the flow which should continue to point out some limitations of trackers as the author does in the next sentence.
8. What is the meaning of the bold part in the first sentence on page 4, column 1: [The article has a **preliminary character** in terms].
9. What or which test stand is being referenced in second paragraph on page 4, column 1, the sentence goes like: [The design of the **test stand**….]
10. This sentence, in column 2, page 4, first paragraph has typos highlighted bold. It also makes a claim of adjustable feet which are not there in the model in the figure number referenced: [….and a set of adjustable feet to give it some room for adjustment of the slope on site. The model and its individual spatial projections **were **shown **of **Figure 2…]
11. The following description, under Test Procedure, must be supported by a bird’s eye view sketch of the site complete with geographical coordinates, otherwise it is unnecessarily complicated [ *Test benches were placed on the furthest side towards the west of the plot and around the middle in the N-S direction. The positioning was dictated by the trees that were growing in the near vicinity of the plot, from the east side. Because of the aforementioned trees, the sun was accessible from around 7:30 CEST (5:30 GMT) in terms of mornings. The view was almost unobstructed from the west side, however, which allowed for tracking the sun almost*...]
12. In the list of steps on page 6, the sentence: [ *activation of test stands around 7:00 CEST (5:00 GMT) and their calibration*] is not clear what was done.
13. The next step too is not clear as to what was exactly done [ *verification of correct solar panel alignment after the calibration and routine check of the state of hardware and software*].
14. What is a research aperture in step 6…[ *stopping the research aperture*…] please show it.
15. Show a diagrammatic illustration or labelled images of your experimental set up so that all the modules mentioned in you procedure such as the one below are clear to the reader…[ *extraction of the gathered data by pulling the SD card with the data from the SD card module and copying the files to the laptop and USB stick for redundancy*].
16. The listed steps will be much better if numbered than just using bullets.
17. The sentence below, immediately after the list of steps, will need editing as the reader will not know what measurement series is, and what samples are.[ *The raw data consisted of the date, at which each measurement series was done, time at which each sample was taken and power readings from the fixed, reference solar panel and the tracking panel*...].
18. First paragraph under results….please attend to the matter of SAMPLES as highlighted in my foregoing comment.
19. Delete the bold part of the sentence below [ *Table 3 shows a difference in energy gathered between tracking panel and a stationary panel, **derived from the data***]
20. With respect to the Tables, I think the [J] in the subtitles is the unit of energy [Joules], if so, the small subtitles for your tables must read like this: [Energy collected in Joules (J)].
21. Results should not include the methodology, please find where to fit the methodology below in the appropriate place under **Test Procedure**. [T *he data were grouped by readings taken from each day of the study period. The day was also divided into three periods: morning (from 7:00 to 10:00), midday (from 10:00 to 15:00) and evening (from 15:00 to 20:00). Throughout the study period, the measurements were taken during different weather conditions. The weather was mostly sunny from 14th to 17th with occasional cloud patches covering the sky from few minutes to about an hour and a short rainfall occurring during the evening of 16th. There was a substantial cloud coverage throughout the evening of 17th.*]
22. Firstly, the author must show, even with simple equations, how the raw measurement inputs (RMI) and calculated interval gains (CIG) percentages were calculated. Secondly, the author must know that the percentages he is proposing lack persuasion as he does not point out to their use by previous researchers, or if they are novel approached, and explain why was a novel approach chosen over existing one. Additionally, my previous comment on unacceptability of putting procedure under results is valid for this sentence/paragraph. The author must find an appropriate place for it under **Test Procedure**.
23. Not clear from the Tables and Figures presented by the author how he comes by this observation [The aggregated data showed a rough increase in tracking panel power generation of about 22–23% in relation to the stationary panel throughout the study period.]
24. How was the Total column in Table 4 calculated?
25.  [… **.was shown on Figure 5**] change to […..is shown in Figure 5].
26. The following claim is not in the paper nor is it part of the data recorded or calculated from recorded data: [ *Results of the study have shown an average daily power output of a stationary solar panel at a level of 0.182 kW-h per day and an average power output of solar tracking panel at a level of 0.224 kW-h per day. y. The difference in outputted power between stationary and solar tracking panel was 0.042 kW-h*…].
27. Look at this sentence in your conclusions, the part I show in bold is not clear [decrease the margin of error that **is contributed to small scale experiments**].
28. Look into the following sentence too and replace with bold work with [by]….[ This is an issue that should also be addressed **before **future studies.]

Is the work clearly and accurately presented and does it cite the current literature?

Partly

Are all the source data underlying the results available to ensure full reproducibility?

Partly

Is the study design appropriate and is the work technically sound?

Partly

Are the conclusions drawn adequately supported by the results?

Partly

Are sufficient details of methods and analysis provided to allow replication by others?

Partly

Reviewer Expertise:

NA

I confirm that I have read this submission and believe that I have an appropriate level of expertise to confirm that it is of an acceptable scientific standard, however I have significant reservations, as outlined above.

# Response

**Published:** 2022-07-12

**Authors:** Jacek Harazin

**Affiliations:** Politechnika Śląska, Poland

Dear Doctor Tjiparuro,

 First and foremost, I’d like to thank you for a very detailed breakdown of your review. It has shed a lot of light on many issues with my article writing. I value that while still being a young researcher, trying to obtain my own title. I hope that my new revision of the article will do justice to your comments. I will try to address your comments in a similar manner.

**Ad1. to Ad4.** Necessary corrections were made

**Ad5.** After reading the paragraph and after some consideration, given that I can now look back at my own work with a dose of criticism, I came to a similar conclusion that there was no more value added in this section and decided to remove it as suggested.

**Ad6.** I have slightly changed the wording of this sentence to point more clearly on panels with solar trackers.

**Ad7.** I have split this section in two and tried to use better wording to present advantages and disadvantages more clearly and avoid contradictions.

**Ad8.** I believe that this sentence was just a product of me being personally a bit too shy and insecure about this area of research. This article was a journey onto new waters for me personally. I have decided to remove this bit of unnecessary digression to avoid confusion.

**Ad9.** This was a reference to the test stand with a solar tracker. I have slightly modified the sentence to make it clearer.

**Ad10.** Yes, that was a mistake of trying to refer to later stages of the project. A set of “screw-on” feet was later added to both test stands to give it more adjustability on the grassy dirt. Unfortunately, I do not have any technical images for the later stages because the project was being developed and readjusted “on the go”. I have tried to describe it in the article itself to correct this mistake.

**Ad11.** I have prepared a sketch, which will be placed as the new “Figure 6”. It is not very technical, but I hope it will be clear enough to give a picture of the testing environment that I have tried to describe previously. A rough geographical position was initially mentioned at the first paragraph under the “Test procedure”. I unfortunately can’t narrow it down any more due to the plot being part of someone’s private property which was graciously lent for our testing purposes.

**Ad12.** I have tried to expand this description and make it more precise.

**Ad13.** This issue has also been addressed similarly.

**Ad14.** I have changed the wording of this point to be more precise of what I operated in that stage. The actual circuit was also added as the new “Figure 5”.

**Ad15.** I have also tried to address this comment with the new figure I’ve placed.

**Ad16.** Numbering has been added.

**Ad17.** I have tried to word this sentence more clearly so it should cause less confusion.

**Ad18.** Same as above.

**Ad19.** The mentioned part was deleted.

**Ad20.** I also changed the naming like you suggested.

**Ad21.** The addressed paragraph was moved to the “Test procedure” section.

**Ad22.** I have also moved this paragraph to the correct section, and I have tried to explain the equations more clearly. I’ve decided to not insert any specific equations because the formula was very basic and simple. I may have not said that in clear enough manner previously. Basically, because my aperture was taking power output measurements only once each 30 seconds (RMI), I figured that CIG may be a helpful datapoint. I have calculated an average amount of energy produced between each consecutive pair of measurements. The average was taken from each two neighbouring power output measurements and then multiplied by the interval of 30 seconds.

**Ad23.** This result was obtained by averaging the registered power output differences and average gathered energy difference between stationary panel and a panel with a solar tracker on the span of the entire experiment (5 days). I have tried to reword this sentence to better explain how these results were obtained.

**Ad24.** The total column in table 4 was calculated in the same way as I have mentioned above, just that the result was based on a daily measurement.

**Ad25.** Correction has been made.

**Ad26.** This claim was a result of a quick conversion of energy gathered by solar panels, mentioned in tables, from Joules into kilo-Watt-hours. I did forget to actually explain where these values came from all of a sudden. I changed the description to make this conclusion less accidental.

**Ad27.** I have tried to rephrase this sentence and make my thoughts more clear.

**Ad28.** The sentence has been corrected.

 Again, thank you very much for your attention to detail. I hope that this set of corrections will be acceptable at the very least. I have tried to improve the clarity of my procedures as much as I could. Unfortunately, I have lost memory of some of my decisions made during this research because of the time it took me to finally address these issues, for which I apologise.

 Best Regards,
